# Study on the Seroprevalence of Cystic Echinococcosis and Toxocariasis in the Rural Population Referred to Reference Laboratory in Urmia, Northwest Iran

**DOI:** 10.1155/japr/8850347

**Published:** 2025-06-06

**Authors:** Fatemeh Ramzi, Rasool Jafari, Elham Yousefi

**Affiliations:** Department of Parasitology and Mycology, School of Medicine, Urmia University of Medical Sciences, Urmia, Iran

**Keywords:** cystic echinococcosis, Iran, toxocariasis

## Abstract

**Introduction:** Cystic echinococcosis (CE) and toxocariasis are the two important zoonoses worldwide, and both are endemic in Iran, especially in rural areas. The present study was aimed at determining the seroprevalence of human CE and toxocariasis in rural inhabitants of Urmia District, Northwest Iran.

**Materials and Methods:** During 6 months (January to May 2023), 698 sera were obtained from the rural population of Urmia District, Northwest Iran, referred to the reference laboratory in Urmia. Seropositivity for CE and toxocariasis was determined in 698 and 430 individuals using enzyme-linked immunosorbent assay kits (ELISA), respectively. Data were analyzed with SPSS v.23 software using respective tests.

**Results:** Eight out of 698 (1.1%) and 87 out of 430 (19.9%) of sera were positive for anti-*Echinococcus* IgG and anti-*Toxocara* IgG, respectively. The CE seropositivity was 1.3% in females compared to 0.8% in males. Anti-*Toxocara* seropositivity was significantly lower in females (46, 17.0%; *p* = 0.036, OR = 0.628) compared to males (41, 24.6%). Furthermore, the seropositivity was significantly higher in patients with a history of consistent dog contact and those who consumed raw vegetables without washing with disinfectants/detergents. A patient was found to be coinfected by both infections, CE and toxocariasis.

**Conclusion:** Based on the results of the present study, both CE and toxocariasis are present in rural areas of Urmia District, which is alarming, because CE is a serious infection even with low prevalence. Toxocariasis, on the other hand, is considerably high in prevalence, showing the high risk of infection with *Toxocara* spp. in these areas.

## 1. Introduction

Zoonotic diseases are concerning regarding human health [1]. Cystic echinococcosis (CE) [2, 3] and toxocariasis [4, 5] are among the important and prevalent zoonotic parasitic diseases worldwide. Both CE [2, 3] and toxocariasis [4, 5] are prevalent among animals and humans in Iran.

Human CE is a parasitic disease caused by a canine tapeworm *Echinococcus granulosus* [6]. CE is a worldwide infection that puts a heavy economic burden on the social well-being of people in several countries. It is endemic in many regions, especially Central Asia, the Middle East, Africa, and South America [7].

In humans, CE is an accidental infection that classically occurs by ingesting *E. granulosus* eggs shed with infected dogs' feces (definitive host) that produce hydatid cysts in the liver, lungs, and other organs of humans (intermediate hosts). CE is characterized by slow-growing hydatid cysts in different organs of the intermediate host [1]. While CE is typically asymptomatic, it causes significant morbidity and occasional mortality with considerable economic losses in livestock and humans [7]. The disease primarily affects humans in rural regions, especially where livestock raising is the main occupation, and egg contamination is highly possible [8].

The signs of CE are location, size, and load-dependent; for instance, the most frequent symptoms of pulmonary CE are coughing, shortness of breath, hemoptysis, atelectasis, retention pneumonia, congestion of the superior vena cava, and in infants, failure to thrive [9]. The signs of hepatic CE are right upper quadrant (hypochondriac) pain, nausea and vomiting, biliary colic, jaundice, hepatomegaly, fistulae, abscesses, ascites, portal hypertension, and inferior vena cava or Budd–Chiari syndromes. Complicated hydatid cysts are almost always symptomatic and are broadly categorized into either rupture and secondary infection or anaphylaxis [10].

Toxocariasis, on the other hand, is a widespread zoonosis with a substantial social and economic impact on different communities around the globe, especially communities suffering from poverty [6]. It is caused by *Toxocara* spp., a roundworm of dogs and to a lesser extent cats. In dogs and cats, which are the natural hosts, *Toxocara* colonizes the intestinal lumen and produces eggs that are shed with feces into the environment. Humans are not suitable for the development of *Toxocara* and are an aberrant [6] or paratenic host [11] that accidentally ingests the eggs or encapsulates juveniles in improperly cooked tissues of paratenic hosts. Consequently, the hatched juveniles cannot become adult worms in the human body [6] and wander in different organs, causing the main clinical syndromes including visceral larva migrans (VLM), ocular larva migrans (OLM), neurologic (NT), and covert or common toxocariasis (CT) [5] when symptoms remain mild and nonspecific. Toxocariasis can also present as pericardial effusion, eosinophilic meningitis, and myocarditis [12]. The wandering juveniles migrate to various human body organs, such as the liver, lungs, kidneys, eyes, heart, brain, and muscle, causing a wide-ranging clinical future. The severity of infection depends on the parasite burden, the extent of juveniles' migration, age, and immune-mediated response [6].

The migrating larvae are attacked by host immune responses, resulting in local inflammation associated with eosinophilia, elevation of cytokines, and production of specific antibodies. VLM is the most common syndrome in humans, manifesting itself by eosinophilia, wheezing, coughing, myalgia, or even cutaneous manifestations such as rash, eczema, pruritus, vasculitis, and panniculitis. Lymphadenopathy, nodules, myocarditis, granulomatous hepatitis, nephritis, arthritis, development of asthma, and promotion of pulmonary fibrosis are also assumed to be linked with VLM [13].

CE is endemic all over Iran, and patients are reported from almost any region. The overall prevalence of CE in Iran was estimated as 5%, which is significantly higher in the north (9%) and west of the country. It is also reported that CE is also higher than average in humans younger than 40 years of age (7%) and the rural population and nomads (6%) [3]. Additionally, human toxocariasis is present in all regions of Iran, and the reports are diverse from 5.3% in Hamadan (2007) [14] to 34.5% in Ahvaz (2009) [15]. Moreover, based on a report, *Toxocara* eggs were found in 75% of the public park's soil in Isfahan, which may be contaminated by stray dogs and cats [16].

Stray dogs and cats are present all around Iran [17]. These animals play important roles in the transmission of toxocariasis to humans, especially in low-income and rural regions [6]. The majority of transmission routes are shared among CE and toxocariasis, and humans become infected by *Toxocara* spp. and *E. granulosus* rather similarly. The present study was aimed at determining the prevalence of CE and toxocariasis in rural inhabitants in Urmia District, Northwest Iran.

## 2. Materials and Methods

### 2.1. Study Region

Urmia County is located in West Azerbaijan Province in the northwest of Iran with a population of 1,040,565. Lake Urmia lies to the east, and the border with Turkey lies to the west of Urmia District. The climate is cold and semiarid.

### 2.2. Sampling

In this cross-sectional study, 698 blood samples were collected from rural inhabitants referring to the reference laboratory of Urmia University of Medical Sciences in Urmia, Northwest Iran, from January to May 2023. Only people from rural areas of the Urmia District at any age and sex were entered into the study. The sera were isolated and kept frozen at −20°C until examination. The samples were evaluated for the presence of IgG antibodies against *E. granulosus*. Among these samples, 438 were examined for IgG antibodies against *Toxocara canis*.

### 2.3. Demographic Variables

A questionnaire with demographic variables and some risk factors, such as sex, age, region, contact with cats and dogs, education, occupation, consumption of local vegetables, washing vegetables, water supply, contact with livestock, animal husbandry, and presence of liver symptoms, was filled out for each volunteer. Then the participants were informed about the study and filled out informed written consent.

### 2.4. Anti-*Echinococcus* IgG Test

Anti-*Echinococcus* IgG was determined using qualitative indirect ELISA kits (Pishtaz-Teb, Iran) to determine the seropositivity against hydatid cysts. The procedure was carried out according to the manufacturer's instructions. The relative sensitivity, specificity, and accuracy of the used ELISA kit were claimed by the company to be 91%, 96%, and 94%, respectively.

### 2.5. Anti-*T. canis* IgG Test

Anti-*T. canis* IgG was determined using a commercial ELISA kit (NovaTec Immundiagnostica GmbH, Germany, Product Number: TOCG0450). The diagnostic specificity and sensitivity of the used kit are claimed to be > 95%. The procedure was carried out according to the manufacturer's instructions. The IgG concentration was also quantified in NovaTec Units (NTUs) using the equation provided by the manufacturer's instructions.

### 2.6. Map

The map of Urmia County was purchased from an online provider (mapme.ir) and modified, and information was added by Photoshop software.

### 2.7. Data Analysis

Data were analyzed by SPSS software (IBM SPSS Statistics for Windows, Version 23.0. Armonk, NY: IBM Corp.) using respective statistics.

## 3. Results

The age range of the participants was 1–99 years with a mean age of 18.452 ± 42.41 (std) years, which all were examined for anti-*Echinococcus* IgG. Eight out of 698 (1.1%) of the collected sera were positive for anti-*Echinococcus* IgG, six (1.3%) were females and two (0.8%) were males. The CE seropositivity was higher in females compared to males (1.3% vs. 0.8%; OR: 1.715), yet it was not statistically significant (Table 1). The number of positive cases was low, and because of that, none of the variables in Table 1 showed a significant relationship.

Out of 698 samples, 438 with a mean age of 18,351 ± 42 and an age range of 1–89 years were examined for IgG against *T. canis*. Considering *Toxocara* seropositivity, 87 out of 438 (19.9%) studied humans showed positive levels of anti-*Toxocara* IgG. Considering sex distribution, *Toxocara* seropositivity was significantly higher in males 41 (24.6%; *p* = 0.036, OR = 1.59) compared to females (46,17.0%). Furthermore, the seropositivity was significantly higher in patients with a history of consistent dog contact (OR = 1.77; *p* = 0.012) and those washed raw consumed vegetables without disinfectants/detergent (*p* < 0.05) (Table 2). A patient was found to be coinfected by both infections, CE and toxocariasis.

The mean age was significantly higher in *Toxocara* seropositive patients compared to seronegative ones (49.28 vs. 39.91 years); however, it was not true for CE (Table 3).

The most affected age group by both toxocariasis and CE was 60–80, which was statistically significant in toxocariasis (*p* ≤ 0.001), yet was not significant in CE (Table 4).

**Figure 1:**
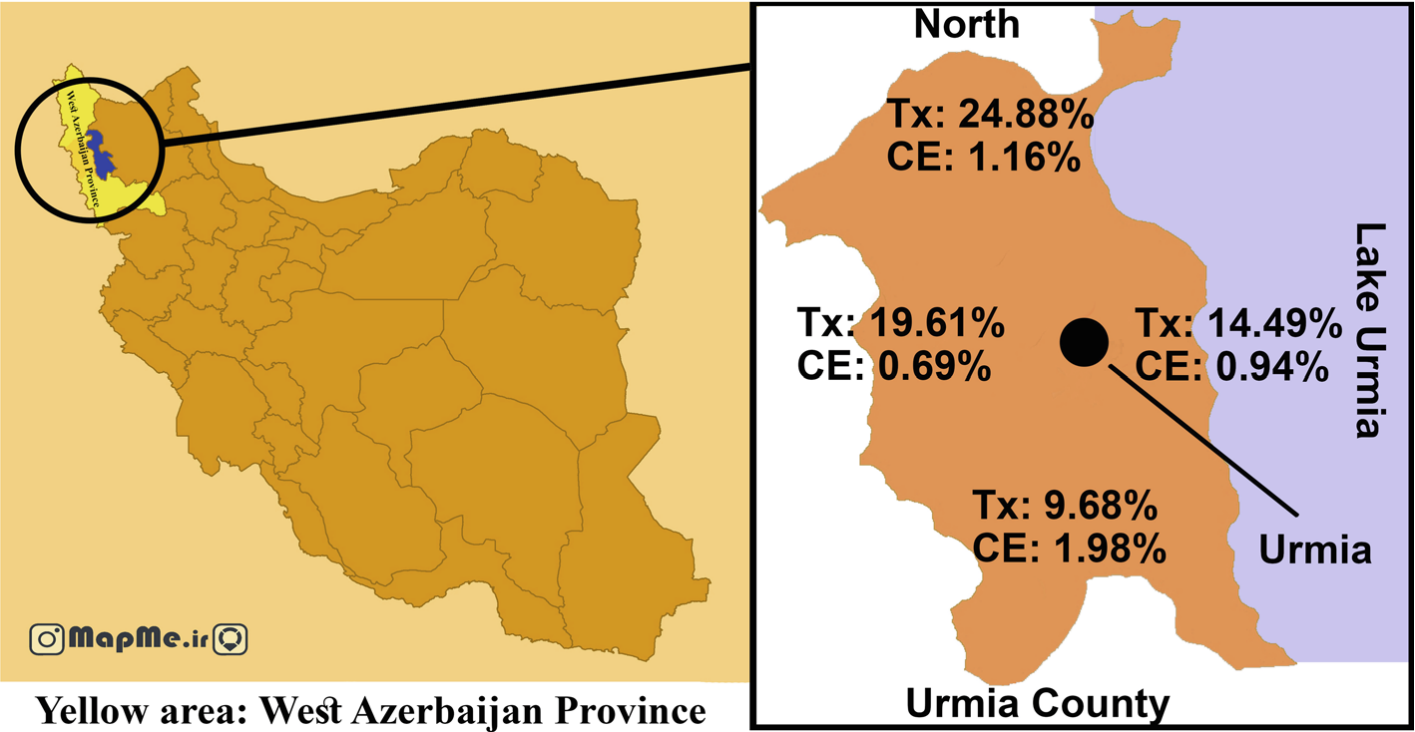
Map of Urmia County in Iran showing seroprevalence of cystic echinococcosis (CE) and toxocariasis (Tx) found in the north, south, west, and east of the county.

The highest seropositivity was found in the north of Urmia District (24.88%) and the lowest in the south (9.68%) (Figure 1). Furthermore, the highest and lowest seropositivity for CE was observed in the rural areas of south and west of Urmia County, respectively (Figure 1).

## 4. Discussion

The present study showed that CE and toxocariasis are present in the rural population of Urmia County, Northwest Iran. The seroprevalence of CE was 1.1%, which may be overlooked, but it is a serious disease. Toxocariasis, on the other hand, is prevalent in the studied area with a considerably high prevalence. Both diseases are zoonotic, and canids are an important source of human infection. Rural populations are in close contact with livestock, dogs, cats, soil/dust particles, or during agriculture, traditionally grazing livestock, slaughtering livestock by themselves with insufficient supervision, and are at particular risk of various infectious agents, especially the eggs of parasitic worms [18–20].

There has been a huge global effort to control CE; however, it remains endemic and even hyperendemic in many countries throughout the world like Iran [21]. Annually, 1.2 million people become affected by CE, and the mortality is estimated at 2.2%. It also results in 3.6 million disability-adjusted life years (DALY) [22]. Khalkhali et al. reported the weighted prevalence of animal and human CE as 15.6% and 4.2%, respectively. The most cases of human CE were reported in the south of Iran (average 5.8%) and lowest in central Iran (average 2.2%) [23]. In the present study, the seroprevalence of human CE in rural areas of Northwest Iran (Urmia County) was found to be 1.1%, which is considerably lower than the average rates reported by Khalkhali et al. Similarly, they reported a higher prevalence of CE in females, which is consistent with our findings in Northwest Iran [23]. Some studies have reported seropositivity in females to be higher than in males [24–26], yet some studies reported the opposite results [27], and a study has reported equal prevalence in males and females [28].

Human CE is commonly reported from healthcare centers in most regions of Iran, and the incidence of human CE has been estimated by Rokni in 2008 at 1.18–3 per 100,000 population [29]. Furthermore, the yearly cost of CE in 2012 is estimated at $93.39 million for the Iranian population [30]. CE is responsible for nearly 1% of admissions to surgical wards, and based on the admission rates, the rate of human infection is estimated at 0.6–1.2/100000 [31], which is lower than the findings of the present study. The reason may be the nature of being asymptomatic in most cases, and not all infected individuals undergo surgery.

Based on the studies, the prevalence of CE in Golestan was 2.34% [32], Kashan was 2.04% [33], Zanjan was 3% [34], Meshginshahr was 1.79% [27], Arak was 3.46% [35], Isfahan was 1.1% [36], Alborz was 3.4% [37], Qom was 1.6% [38], nomads of Southern Iran were 13.7% [32], Sanandaj was 7.3% [39], Ilam was 1.2% [40], and Tehran was 1.63% [41]. The highest and lowest prevalence was reported for the northeast (15.2%) and southeast (0.7%) of Iran, respectively [21]. The found seroprevalence in the present study was 1.1%, which is more or less similar to other parts of Iran, especially Isfahan Province [36]. Furthermore, the highest and lowest seropositivity for CE in the present study was observed in the rural areas of south and west of Urmia County, respectively.

In the present study, a considerably high proportion (19.9%) of studied rural inhabitants was seropositive for toxocariasis, which is alarming. In a systematic review, the overall seroprevalence of toxocariasis in Iran is estimated at 6.58% [42].

There are some limitations of the *T. canis* serological test: one is the cross-reactivity with other worms causing human infections, such as *Ascaris lumbricoides*, particularly in endemic areas. The other is the long-lasting serum IgG level that can remain high for years. Nevertheless, there is a potential false-positive reaction, yet the *Toxocara* IgG assays have clinical significance that should not be ignored [6]. In Iran, intestinal helminthic infections were very high in the past; however, nowadays, they are rare and seldom reported in studies in the country [43].

A systematic review and meta-analysis by Rostami et al. estimated the seroprevalence of human toxocariasis in the world as 19% (16.6%–21.4%) [44], which is close to the results of the present study in the Urmia area. In the meta-analysis study by Eslahi et al., the weighted mean prevalence of human toxocariasis in Iran was estimated to be 9.3% (6.3%–13.1%) [4], considerably lower than our results in Urmia. The prevalence of human toxocariasis in various regions of Iran is reported in Chaharmahal Bakhtiari and Hamedan (5.3%), Shiraz (25.6%), Kermanshah (8.5%), Zanjan (2.7%), Ahvaz (34.5%), a neighboring city Tabriz (29.3%), Tehran (6.2%), Mashhad (20.4%), and Arak (1.8%) [5]. Mazandaran, East Azerbaijan, and Fars provinces had the highest seroprevalence [45–47]. These areas may have a favorable climate for the survival of the eggs of the parasite [47].

In the present study, *Toxocara* seropositivity was significantly higher in females, humans with a history of consistent dog contact, and those who washed raw consumed vegetables without disinfectants/detergents. Furthermore, by increasing age, the seroprevalence also increases, and the highest rate was found in the 60–80-year-old age group. The highest and lowest seropositivity for toxocariasis was observed in the humans referred from rural areas of north and south of Urmia County, respectively (Figure 1). The latter shows a higher risk of toxocariasis in northern Urmia County.

## 5. Conclusion

According to the findings of the present study, CE and toxocariasis are common in the rural areas of Urmia County, Northwest Iran. Due to the dangerous nature of CE, its low prevalence is also important. Toxocariasis has a significantly high prevalence in the rural areas of Urmia, especially the northern regions of the county, which require measures such as fighting reservoirs and healthcare education.

## 6. Limitations of the Study

We had two main limitations: first, we could not confirm the CE results by ultrasound, and second, we could not confirm toxocariasis by western blot.

## Figures and Tables

**Table 1:** CE seropositivity in the studied humans from Urmia District considering different demographics and risk factors.

**Variable**		**CE**		**OR**	**Total**	**p**	**95% CI**
		**Positive**	**Negative**				
Sex	Female	6 (1.3%)	439 (98.7%)	1.715	445 (100%)	0.398	0.344–8.562
	Male	2 (0.8%)	251 (99.2%)	1	253 (100%)		—

Level of education	Diploma or higher	0 (0.0%)	119 (100%)	0	119 (100%)	0.996	0
	High school	0 (0.0%)	7 4 (100%)	0	74 (100%)	0.997	0
	Elementary	1 (0.6%)	168 (99.4%)	0.167	169 (100%)	0.210	0.01–2.742
	Illiterate	6 (2.0%)	301 (98.0%)	0.558	307 (100%)	0.595	0.065–4.802
	Guidance school^a^	1 (3.4%)	28 (96.6%)	1	29 (100%)	0.786	—

Occupation	Unemployed/housewife^a^	7 (1.5%)	449 (98.5%)	1	456 (100%)	0.972	—
	Worker	0 (0.0%)	11 (100%)	0	11 (100%)	0.999	0
	Farmer	1 (0.1%)	108 (99.1%)	0.594	109 (100%)	0.628	0.072–4.878
	Self-employed	0 (0.0%)	122 (100%)	0	122 (100%)	0.996	0

Dog contact	Yes	3 (1.0%)	284 (99.0%)	0.858	287 (100%)	0.568	0.203–3.618
	No	5 (1.2%)	406 (98.8%)	1	411 (100%)		—

Livestock contact	Yes	2 (0.8%)	238 (99.2%)	0.633	240 (100%)	0.442	0.127–3.161
	No	6 (1.3%)	452 (98.7%)	1	458 (100%)		—

Vegetable	Bought	3 (2.4%)	123 (97.6%)	2.766	126 (100%)	0.161	0.652–11.727
	Self-cultured	5 (0.9%)	567 (99.1%)	1	572 (100%)		—

Water	Tap water	7 (1.1%)	612 (98%)	0.41	619 (100%)	0.412	0.049–3.438
	Spring water	0 (0.0%)	42 (100%)	0	42 (100%)	0.998	0
	Well water^a^	1 (2.7%)	36 (97.3%)	1	37 (100%)	0.715	—

Vegetable wash	Salt and water	4 (1.4%)	273 (98.6%)	3.57	277 (100%)	0.256	0.397–32.204
	Detergent	2 (2.2%)	88 (97.8%)	5.54	90 (100%)	0.164	0.497–61.916
	Vinegar	1 (1.2%)	85 (98.8%)	2.87	86 (100%)	0.458	0.178–46.400
	Water^a^	1 (0.4%)	244 (99.6%)	1	245 (100%)	0.574	—

Signs	URQ pain	0 (0.0%)	29 (100%)	0	29 (100%)	0.998	0
	Chest pain	1 (3.4%)	29 (96.6%)	3.23	29 (100%)	0.281	0.384–27.155
	No sign^a^	7 (1.1%)	633 (98.9%)	1	640 (100%)	0.559	—

Soil contact	Yes	1 (0.8%)	119 (99%)	0.685	120 (100%)	0.588	0.084–5.623
	No	7 (1.2%)	517 (98.8%)	1	578 (100%)		—

Total		8 (1.1%)	690 (98.9%)		698 (100%)		

^a^Constant variable (indicator).

**Table 2:** Anti-*Toxocara* seropositivity in 438 studied humans considering different demographics and risk factors.

**Variable**		**Toxocariasis**		**OR**	**Total**	**p**	**95% CI for OR**
		**Positive**	**Negative**				
Sex	Female	46 (17.0%)	225 (83.0%)	0.628	271 (100%)	0.036	0.391–1.009
	Male	41 (24.6%)	126 (75.4%)	1	167 (100%)		—

Level of education	Diploma or higher^a^	10 (11.6%)	76 (88.4%)	1	86 (100%)	0.072	—
	High school	9 (16.4%)	46 (83.6%)	1.48	55 (100%)	0.424	0.562–3.931
	Elementary	21 (20.6%)	81 (79.4%)	1.97	102 (100%)	0.103	0.872–4.454
	Illiterate	46 (25.4%)	135 (74.6%)	2.59	181 (100%)	0.012	1.236–5.424
	Guidance school	1 (7.1%)	13 (92.2%)	0.58	14 (100%)	0.623	0.069–4.960

Occupation	Unemployed/housewife^a^	47 (17%)	230 (83%)	1	277 (100%)	0.122	—
	Worker	3 (42.9%)	4 (57.1%)	3.67	7 (100%)	0.096	0.795–16.941
	Farmer	19 (26.8%)	52 (73.2%)	1.79	71 (100%)	0.063	0.970–3.297
	Self-employed	18 (21.7%)	65 (78.3%)	1.35	83 (100%)	0.328	0.737–2.492

Dog contact	Yes	46 (25.3%)	136 (74.7%)	1.77	128 (100%)	0.012	1.106–2.845
	No	41 (16.0%)	215 (84.0%)	1	256 (100%)		—

Cat contact	Yes	11 (24.4%)	34 (75.6%)	1.35	45 (100%)	0.263	0654–2.785
	No	76 (19.3%)	317 (80.7%)	1	393 (100%)		—

Livestock contact	Yes	35 (23.6%)	113 (76.4%)	1.42	148 (100%)	0.099	0.874–2.299
	No	52 (17.9%)	238 (82.2%)	1	290 (100%)		—

Vegetable	Bought	18 (20.0%)	72 (80.0%)	1.01	90 (100%)	0.537	0.566–1.805
	Self-cultured	69 (19.8%)	279 (80.2%)	1	348 (100%)		—

Water	Tap water	80 (20.9%)	303 (79.1%)	1	383 (100%)	0.331	—
	Spring water	3 (10.0%)	27 (90.0%)	0.42	30 (100%)	0.164	0.124–1.423
	Well water	4 (16.0%)	21 (84.0%)	0.72	25 (100%)	0.56	0.221–2.161

Vegetables wash	Salt and water	33 (19.6%)	135 (80.4%)	0.84	167 (100%)	0.063	0.495–1.423
	Detergent	3 (5.8%)	49 (94.2%)	0.21	52 (100%)	0.515	0.062–0.713
	Vinegar	14 (25.9%)	40 (74.1%)	1.2	54 (100%)	0.012	0.590–2.444
	Water^a^	37 (22.6%)	127 (77.4%)	1	164 (100%)	0.613	—

Signs	URQ pain	6 (26.1%)	17 (73.9%)	1.5	23 (100%)	0.410	0.572–3.932
	Chest pain	5 (31.3%)	11 (68.8%)	1.93	16 (100%)	0.235	0.652–5.724
	No sign^a^	76 (19.0%)	323 (81%)	1	399 (100%)	0.371	—

Soil contact	Yes	22 (28.2%)	56 (71.8%)	1.78	78 (100%)	0.042	1.017–3.126
	No	65 (18.1%)	295 (81.9%)	1	360 (100%)		—

Total		87 (19.9%)	351 (80.1%)		438 (100%)	438	—

^a^Constant variable (indicator).

**Table 3:** Mean age comparison among CE and toxocariasis seropositive and seronegative cases.

**Infection**		**N**	**Mean**	**Std. deviation**	**Mean rank**	**p**
Toxocariasis	Positive	87	49.28	15.363	274.28	< 0.001
	Negative	351	39.91	18.574	205.92	
CE	Positive	8	45.13	26.079	397.63	0.497
	Negative	690	42.38	18.370	348.94	

**Table 4:** CE and toxocariasis seropositivity in different age groups.

**Age groups**	**Toxocariasis**		**Total**	**OR**	**95% CI**	**p**	**CE**		**Total**	**OR**	**95% CI**	**p**
	**Positive**	**Negative**					**Positive**	**Negative**				
0–20^a^	3 (5.4%)	53 (94.6%)	56	1		0.002	2 (2.3%)	86 (97.7%)	88	1	—	0.259
20–40	21 (14.5%)	124 (85.5%)	145	2.992	0.856–10.461	0.086	1 (0.5%)	215 (99.5%)	216	0.2	0.018–2.234	0.191
40–60	38 (25.2%)	113 (74.8%)	151	5.941	1.754–20.120	0.004	1 (0.4%)	251 (99.6%)	252	0.17	0.015–1.913	0.152
60–80	25 (30.9%)	56 (69.1%)	81	7.887	2.248–27.668	0.001	4 (3%)	131 (97%)	135	1.31	0.235–7.325	0.756
> 80	0 (0%)	5 (100%)	5	0	0	0.999	0 (0%)	7 (100%)	7	0	0	0.999
Total	87 (19.9%)	351 (80.1%)	438				8 (1.1%)	690 (98.9%)	698			

^a^Constant variable (indicator).

## Data Availability

The datasets, raw or analyzed in SPSS format, will be available from the corresponding author upon reasonable request.
